# Neuroimaging genomics in psychiatry—a translational approach

**DOI:** 10.1186/s13073-017-0496-z

**Published:** 2017-11-27

**Authors:** Mary S. Mufford, Dan J. Stein, Shareefa Dalvie, Nynke A. Groenewold, Paul M. Thompson, Neda Jahanshad

**Affiliations:** 1 UCT/MRC Human Genetics Research Unit, Division of Human Genetics, Department of Pathology, Institute of Infectious Disease and Molecular Medicine, Faculty of Health Sciences, University of Cape Town, Cape Town, South Africa 7925; 2 MRC Unit on Risk and Resilience, Faculty of Health Sciences, University of Cape Town, Cape Town, South Africa 7925; 3 Department of Psychiatry and Mental Health, Groote Schuur Hospital, Cape Town, South Africa 7925; 4 Department of Psychiatry and Mental Health, University of Cape Town, Cape Town, South Africa 7925; 5 Imaging Genetics Center, Mark and Mary Stevens Neuroimaging and Informatics Institute, Keck School of Medicine of the University of Southern California, Los Angeles, CA 90292 USA

## Abstract

Neuroimaging genomics is a relatively new field focused on integrating genomic and imaging data in order to investigate the mechanisms underlying brain phenotypes and neuropsychiatric disorders. While early work in neuroimaging genomics focused on mapping the associations of candidate gene variants with neuroimaging measures in small cohorts, the lack of reproducible results inspired better-powered and unbiased large-scale approaches. Notably, genome-wide association studies (GWAS) of brain imaging in thousands of individuals around the world have led to a range of promising findings. Extensions of such approaches are now addressing epigenetics, gene–gene epistasis, and gene–environment interactions, not only in brain structure, but also in brain function. Complementary developments in systems biology might facilitate the translation of findings from basic neuroscience and neuroimaging genomics to clinical practice. Here, we review recent approaches in neuroimaging genomics—we highlight the latest discoveries, discuss advantages and limitations of current approaches, and consider directions by which the field can move forward to shed light on brain disorders.

## Background

Neuroimaging genomics is a relatively new and rapidly evolving field that integrates brain imaging and individual-level genetic data to investigate the genetic risk factors shaping variations in brain phenotypes. Although this covers a broad range of research, one of the most important aims of the field is to improve understanding of the genetic and neurobiological mechanisms underlying various aspects of neuropsychiatric disorders—from symptoms and etiology, to prognosis and treatment. The goal is to identify key components in biological pathways that can be evaluated or monitored to improve diagnostic and prognostic assessments, and that can ultimately be targeted by novel therapies.

Broadly speaking, existing brain imaging methods can be divided into those that provide data on structure—for example, computed tomography (CT), structural magnetic resonance imaging (MRI), and diffusion–tensor imaging (DTI); function—for example, functional MRI (fMRI), arterial spin labeling (ASL); and molecular imaging—for example, single-photon emission computed tomography (SPECT) and positron-emission tomography (PET) using receptor-binding ligands and magnetic resonance spectroscopy (MRS) [1]. A range of additional new methods have become available for animal and/or human brain imaging, including optical imaging, cranial ultrasound, and magnetoencephalography (MEG), but to date these have been less widely studied in relation to genomics. Future work in imaging genomics will rely on further advances in neuroimaging technology, as well as on multi-modal approaches.

**Fig. 1:**
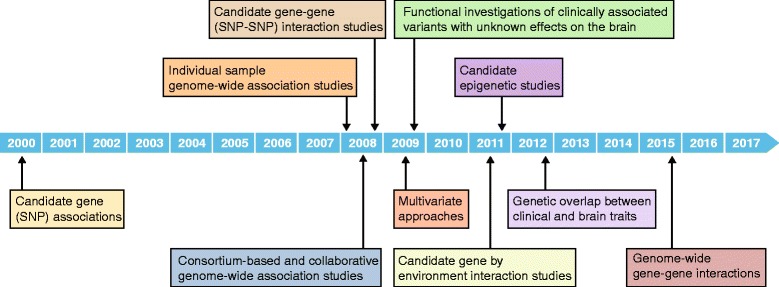
Timeline of methodological approaches common in neuroimaging-genomics studies of neuropsychological disorders. The field of neuroimaging genomics was initiated in the early 2000s using a hypothesis-driven candidate-gene approach to investigate brain and behavior phenotypes [2, 3]. Towards the end of the decade, other candidate-gene approaches, investigating alternative genetic models, began to emerge. These included gene–gene interactions [172], gene–environment interactions [7], and epigenetic effects [6]. Simultaneously, hypothesis-free approaches such as genome-wide association studies (GWAS) were initiated [173] and the need for increased statistical power to detect variants of small individual effects soon led to the formation of large-scale consortia and collaborations [36, 37]. The emergence of the “big data” era presented many statistical challenges and drove the development of multivariate approaches to account for these [174]. GWAS of neuropsychological disorders soon identified significant associations with genetic variants with unknown biological roles, resulting in candidate neuroimaging genomics studies to investigate and validate the genetic effects on brain phenotypes [175]. The emergent polygenic nature of these traits encouraged the development of polygenic models and strategies to leverage this for increased power in genetic-overlap studies between clinical and brain phenotypes [114]. Most recently, hypothesis-free approaches are starting to extend to alternative genetic models, such as gene–gene interactions [70]

Progress in both neuroimaging and genomic methods has contributed to important advances—from candidate-gene (or more precisely, single-variant) approaches initiated almost two decades ago [2, 3], to recent breakthroughs made by global collaborations focused on GWAS [4], gene–gene effects [5], epigenetic findings [6], and gene–environment interactions [7] (Fig. 1). Developments in the field of neuroimaging genomics have only recently begun to provide biological insights through replicated findings and overlapping links to disease—we now know the field holds much promise, but further work and developments are needed to translate findings from neuroimaging genomics into clinical practice. In this review, we discuss the most recent work in neuroimaging genomics, highlighting progress and pitfalls, and discussing the advantages and limitations of the different approaches and methods now used in this field.

## Heritability estimates and candidate gene associations with imaging-derived traits

Approximately two decades ago, neuroimaging genomics had its inception—twin and family designs from population genetics were used to calculate heritability estimates for neuroimaging-derived measures, such as brain volume [8], shape [9, 10], activity [11], connectivity [12], and white-matter microstructure [13]. For almost all these imaging-derived brain measures, monozygotic twin pairs showed greater correlations than dizygotic twins, who in turn showed greater correlations than more-distant relatives and unrelated individuals. These studies confirm that brain measures derived from non-invasive scans have a moderate to strong genetic underpinning [14, 15] and open the doors for more-targeted investigations. These brain features might now be considered useful endophenotypes (using only certain symptoms—for example, altered brain volume—of a trait such as schizophrenia, which might have a more-robust genetic underpinning) for psychiatric disorders [16]. A focus on the underlying mechanisms is central to the now highly regarded Research Domain Criteria (RDoC) research framework [17]. In contrast to classifications that focus on diagnoses or categories of disorders [18, 19], RDoC emphasizes transdiagnostic mechanisms (investigating overlapping symptoms across diagnoses) that emerge from translational neuroscience [20].

Early imaging genomics work (from approximately 2000 to 2010; Fig. 1) focused predominantly on candidate-gene approaches—in the absence of large GWAS datasets, investigators relied on biological knowledge to develop hypotheses. Genetic variants or single-nucleotide polymorphisms (SNPs) identified through linkage studies or located near or within genes with putative biological roles, particularly those involved in neurotransmission, were investigated in brain imaging studies. Early candidate genes studied in relation to brain phenotypes included the sodium-dependent serotonin transporter gene (*SLC6A4*) in individuals with anxiety and depression [21–23] and the catechol-O-methyltransferase gene (*COMT*) in individuals with schizophrenia [24–28].

A key criticism of this early work was that candidate-gene studies were insufficiently powered, with the possibility that small false-positive studies were being published, whereas larger negative analyses were being “filed away” [29, 30]. In support of this view, several meta-analyses have emphasized the inconsistency of small candidate-gene studies [31–33]. These studies noted that, given relatively small effect sizes, larger studies were needed and that a clear focus on harmonization of methods across studies was needed for meaningful meta-analyses. For example, a meta-analysis of candidate studies of the rs25532 polymorphism of *SLC6A4* (commonly referred to as the “short variation”) and amygdala activation, which incorporated unpublished data, was unable to identify a significant association [31]. This finding cast doubt on the representativeness of effect sizes reported in early studies with positive findings, highlighting a potential “winner’s curse” and emphasized the importance of publication bias in the field.

However, borrowing strategic approaches from studies of anthropometric traits (GIANT consortium), psychiatric disorders (PGC, psychiatric genomics consortium [34]), cancer (CGC, cancer genomics consortium [35]), and cardiovascular health and aging (CHARGE [36]), the imaging-genomics community has built large-scale collaborations and consortia in order to obtain the statistical power necessary to disentangle the genetic architecture of brain phenotypes [37].

## Genome-wide association studies in imaging genomics

Imaging genomics has increasingly moved towards a GWAS approach, using large-scale collaborations to improve power for the detection of variants with small independent effects [29]. Examples of such consortia include the Enhancing Neuro-imaging through Meta-analysis (ENIGMA) consortium [37], Cohorts for Heart and Aging Research in Genomic Epidemiology (CHARGE) consortium [36], Alzheimer's Disease Neuroimaging Initiative (ADNI), IMAGEN, which is focused on adolescents [38], and the Uniform Neuro-Imaging of Virchow-Robin Spaces Enlargement (UNIVRSE) consortium [39]. The growing number of GWAS of brain phenotypes and of neuropsychiatric disorders has, on occasion, lent support to previously reported candidate variants [40], but importantly has identified many new variants of interest [41].

An early study by the ENIGMA consortium consisted of approximately 8000 participants, including healthy controls and cases with psychiatric disorders [42]. This study identified significant associations between intracranial volume and a high-mobility group AT-hook 2 (*HMGA2*) polymorphism (rs10784502), and between hippocampal volume and an intergenic variant (rs7294919). A subsequent collaboration with the CHARGE consortium, including over 9000 participants, replicated the association between hippocampal volume and rs7294919, as well as identifying another significant association with rs17178006 [43]. In addition, this collaboration has further validated and identified other variants associated with hippocampal volume [44] and intracranial volume [45], with cohorts of over 35,000 and 37,000 participants, respectively. Another analysis of several subcortical volumes (ENIGMA2), with approximately 30,000 participants, identified a significant association with a novel intergenic variant (rs945270) and the volume of the putamen, a subcortical structure of the basal ganglia [4]. More recently, a meta-analysis of GWAS of subcortical brain structures from ENIGMA, CHARGE, and the United Kingdom Biobank was conducted [46]. This study claims to identify 25 variants (20 novel) significantly associated with the volumes of the nucleus accumbens, amygdala, brainstem, caudate nucleus, globus pallidus, putamen, and thalamus amongst 40,000 participants (see the “Emerging pathways” section later for a more detailed discussion). Moreover, many large-scale analyses [15, 46] are now first being distributed through preprint servers and social media. In another example, in over 9000 participants from the UK Biobank, Elliot and colleagues [15] used six different imaging modalities to perform a GWAS of more than 3000 imaging-derived phenotypes, and identified statistically significant heritability estimates for most of these traits and implicated numerous associated single-nucleotide polymorphisms (SNPs) [15]. Such works still need to undergo rigorous peer-review and maintain strict replication standards for a full understanding of findings, yet this work highlights the fact that the depth of possibilities now available within the field of neuroimaging genomics appears to be outpacing the current rate of publications. As of November 2017, ENIGMA is currently undertaking GWAS of the change in regional brain volumes over time (ENIGMA-Plasticity), cortical thickness and surface area (ENIGMA-3), white-matter microstructure (ENIGMA-DTI), and brain function as measured by EEG (ENIGMA-EEG).

Although neuroimaging measurements only indirectly reflect the underlying biology of the brain, they remain useful for in vivo validation of genes implicated in GWAS and lend insight into their biological significance. For example, the rs1006737 polymorphism in the gene encoding voltage-dependent L-type calcium channel subunit alpha-1C (*CACNA1C*) was identified in early GWAS of bipolar disorder [47, 48] and schizophrenia [49, 50], but its biology was unknown. Imaging-genomics studies of healthy controls and individuals with schizophrenia attempted to explain the underlying biological mechanisms. Studies reported associations of this variant with increased expression in the human brain, altered hippocampal activity during emotional processing, increased prefrontal activity during executive cognition, and impaired working memory during the n-back task [51–53], a series of task-based assessments relying on recognition memory capacity. As the psychiatric genomics field advances and more reliable and reproducible genetic risk factors are identified, imaging genomics will continue to help understand the underlying biology.

The limitations of GWAS of complex traits and neuropsychiatric disorders deserve acknowledgment. In particular, although GWAS can identify statistically significant associations, these have particularly small individual effect sizes and, even cumulatively, do not account for a substantial fraction of the heritability of the relevant phenotype estimated from family models [54]. Furthermore, many associated variants are currently not functionally annotated and most often are found in non-coding regions of the genome, which are not always well understood [55, 56]. Increasing power, through increasing sample sizes, will likely implicate additional variants, but these might not necessarily play a directly causal role [57]. This could be because of the small effect sizes of causative variants, linkage disequilibrium with other variants, and the indirect effects of other variants in highly interconnected pathways [57]. Currently, most studies utilize participants of European ancestry, and replication studies using alternative ethnic groups are required for further discovery and validation of significant associations, which might be influenced by the populations under investigation [58]. Thus, additional strategies are needed to understand fully the genetic architecture of brain phenotypes and neuropsychiatric disorders. These methods can be summarized into three categories: first, delving deeper into rarer genetic variations; second, incorporating models of interactions; and, third, investigating more than a single locus and instead expanding to incorporate aggregate or multivariate effects; these methods and more are discussed below [57].

## Copy-number variation and brain variability

Growing recognition of the neuropsychiatric and developmental abnormalities that arise from rare genetic conditions, such as 22q11 deletion syndrome [59], has led imaging-genomic studies to further explore the relationships between copy-number variations (CNVs) and neural phenotypes [60–63]. For example, in a recent large-scale study of over 700 individuals, 71 individuals with a deletion at 15q11.2 were studied to examine the effects of the genetic deletion on cognitive variables [60]. These individuals also underwent brain MRI scans to determine the patterns of altered brain structure and function in those with the genetic deletion. This study identified significant associations between this CNV and combined dyslexia and dyscalculia, and with a smaller left fusiform gyrus and altered activation in the left fusiform and angular gyri (regions in the temporal and parietal lobes of the brain, respectively). Another study investigating the 16p11.2 CNV, with established associations with schizophrenia and autism, found that the CNVs modulated brain networks associated with established patterns of brain differences seen in patients with clinical diagnoses of schizophrenia or autism [61]. These studies indicate that CNVs might play an important role in neural phenotypes, and initiatives such as ENIGMA-CNV [63] aim to explore this further.

## Gene–gene interactions

Gene–gene interactions (epistasis), where the phenotypic effect of one locus is affected by the genotype(s) of another, can also play significant roles in the biology of psychiatric disorders [64]; such interactions might help account for the missing heritability observed with genetic association testing [54]. Singe-locus tests and GWAS might not detect these interactions as they use additive genetic models [64]. The inclusion of interaction tests has also, for example, been shown to improve the power for detection of the main effects in type 1 diabetes [65]. Recently, this has emerged as a focus of imaging-genomic studies, predominantly using a candidate-gene approach [66–69].

Studies of epistasis are, however, at an early stage and currently have relatively small sample sizes and lack replication attempts, limiting the validity of these findings [70]. Selecting candidate genes for investigation, usually based on significance in previous association studies, may miss important interactions with large effects [71]. Genome-wide interaction approaches may provide for a more unbiased approach towards understanding epistatic effects. As a proof of concept, one such study investigated genome wide SNP–SNP interactions using participants from the ADNI cohort, and the Queensland Twin Imaging study for replication [70]. While larger scale studies are needed to confirm specific findings, this study identified a significant association between a single SNP–SNP interaction and temporal lobe volume, which accounted for an additional 2% of the variance in temporal lobe volume (additional to the main effects of SNPs) [70]. As the power for GWAS in imaging genomics increases through growing consortia and biobanks, large-scale epistatic studies may become possible and explain more of the genetic variance underlying brain structure and function.

## Gene–environment interactions

Most neuropsychiatric disorders have a multifactorial etiology [72, 73], with varying heritability estimates under different conditions [74]. Imaging-genomics studies have begun to investigate how genes and the environment interact (GxE) to influence brain structure and function in relation to neuropsychiatric disorders [75]. These interactions are of further interest as emerging evidence indicates that some individuals exposed to certain environmental factors have altered treatment responses [75]. For example, GxE studies of the rs25532 polymorphism within the *SLC6A4* gene indicate that carriers with depression, and who are exposed to recent life stressors, respond poorly to treatment with certain antidepressants [76–79], but have better responses to psychotherapy compared to those with the alternative genotype [80]. Therefore, imaging genomics is ideally suited to identify possible interactions that may affect treatment responses, lend insight into these mechanisms potentially leading to altered or new therapeutic regimens, and identify at-risk individuals who may benefit from early interventions [81, 82].

Small exploratory studies have suggested that potentially interesting gene–gene interactions might exist [7, 83–89]; however, the statistical power of published analyses is low, and replication is key [90, 91]. Candidate-gene approaches to GxE studies have been commonplace, but these might oversimplify genetic models, as each of these variants contributes minimally to disease risk [90, 91]. To ensure the effect is indeed an interaction and not due to one component of the interaction, all terms (G, E, GxE) will need to be included in a regression model. Naturally, this implies genome-wide interaction studies would require even larger sample sizes than GWAS if they are to be appropriately powered [90, 91]. Concerns about the measures of both phenotype and the exposome (lifetime environmental exposures) have also been raised, as studies using different measures and at different stages of life can produce conflicting results [91–93]. Large-scale collaborations using carefully harmonized protocols will likely be able to mitigate these limitations.

## Epigenetics

Approaches investigating the associations between epigenetic alterations and brain measures once again began with candidate genes [94, 95]. However, disparities between the methylation states of blood, saliva, and brain tissue remain important limitations for untangling the discrepancies found with epigenetic studies [96]. To illustrate this, several projects, such as the Human Roadmap Epigenomics project [97], the International Human Epigenome Consortium [98], and Braincloud [99], have begun developing reference epigenomes, which could pave the way for harmonizing and pooling data across independent datasets. These projects might also provide new biologically based candidates for research—it has been suggested that genes most similarly methylated between blood and brain tissue be investigated first in neuroimaging studies [100, 101]. Recently, imaging consortia such as ENIGMA have begun epigenome-wide association studies for key brain measures such as hippocampal volume, revealing promising associations [102]. Longitudinal and trans-generational studies of both healthy and at-risk individuals might also prove useful for understanding the impact of the environment on the epigenome [101].

## Mapping the genetic structure of psychiatric disease onto brain circuitry

Recent large-scale GWAS of psychiatric disorders have begun to identify significantly associated variants [41, 103]—however, the effect sizes of these variants are small (usually less than 1%) and do not account for the predicted heritability of these traits (as high as 64–80% in schizophrenia [104, 105]). It is hypothesized that many psychiatric disorders have a polygenic (effected by multiple genetic variants) and heterogeneous (disease-causing variants can differ between affected individuals) genetic architecture, resulting in a failure to reach statistical significance and contributing to the phenomenon of missing heritability [106]. GWAS of subcortical brain structure and cortical surface area have also started to reveal significant genetic associations and a polygenic etiology [44–46, 107], although the extent of polygenicity appears to be less than that predicted for psychiatric disorders [107]. Recent studies have begun to disentangle whether the genetics of brain phenotypes overlap with that of psychiatric disorders by making use of their polygenic nature [108, 109].

Polygenic risk scoring (PRS) is one such analytical technique that exploits the polygenic nature of complex traits by generating a weighted sum of associated variants [106, 110, 111]. PRS uses variants of small effect (with *p* values below a given threshold), identified in a GWAS from a discovery dataset to predict disease status for each participant in an independent replication dataset [111]. In large-scale GWAS of schizophrenia, for example, the PRS now accounts for 18% of the variance observed [41]. PRS in imaging genomics has the potential advantage of addressing many confounders, such as the effects of medication and the disease itself through investigation of unaffected and at-risk individuals [112, 113]. For example, PRS for major depressive disorder (MDD; *n* = 18,749) has been associated with reduced cortical thickness in the left amygdala-medial prefrontal circuitry among healthy individuals (*n* = 438) of European descent [114].

However, as with other approaches, PRS is not without limitations. For example, an additive model of variant effects is assumed, disregarding potentially more-complex genetic interactions [115]. The predictive capacity of PRS is also largely dependent on the size of the discovery dataset (ideally greater than 2000 individuals), which is likely still underpowered in many instances [106]. Furthermore, PRS does not provide proportionate weight to biologically relevant genes for neural phenotypes as it is also subject to the confounding elements of GWAS emphasized earlier [57, 113, 116]. Thus, other approaches such as linkage disequilibrium score regression for genetic correlation (a technique that uses GWAS summary statistics to estimate the degree of genetic overlap between traits) [117], Bayesian-type analyses [118], and biologically informed multilocus profile scoring [119, 120] might be alternatives worth exploring, perhaps in conjunction with PRS [121]. More recently, an omnigenic model has been proposed—which takes into account the interconnected nature of cellular regulatory networks that can confound other polygenic models [57].

Linkage-disequilibrium score regression [117] did not identify genetic overlap between schizophrenia (33,636 cases, 43,008 controls) and subcortical volumes (*n* = 11,840 healthy controls), but provided a useful proof-of-principle of this approach [108]. A partitioning-based heritability analysis [122], which estimates the variance explained by all the SNPs on a chromosome or the whole genome rather than testing the association of particular SNPs with the trait, indicated that variants associated with schizophrenia (*n* = 1750) overlapped with eight brain structural phenotypes, including intracranial volume and superior frontal gyrus thickness [109]. Publicly available GWAS data for several other psychiatric disorders were also investigated and indicated that intracranial volume was enriched for variants associated with autism spectrum disorder (ASD), and right temporal pole surface area was enriched for variants associated with MDD, and left entorhinal cortex thickness showed enrichment for bipolar disorder risk variants [109]. These types of analyses confirm a common genetic basis between risk for altered brain structure and neuropsychiatric disorders [16].

## Multivariate approaches

To explain more of the variance in gene-imaging findings, techniques for data-driven discovery using multivariate approaches have begun to emerge in this field. These techniques include methods such as independent component analysis (ICA) [123], canonical correlation analysis [124], sparse partial least squares [125], and sparse reduced-rank regression [126]. To date, the increased explanatory power provided by these approaches has mainly been shown in single datasets or relatively small studies—these often claim to identify significant associations at a genome-wide level [127–129]. Owing to the large number of input variables and parameters (many dimensions), often paired with limited data-points and split-sample training and testing from the same cohort, there can be concerns about overfitting and models that do not generalize. Thus, dimensionality reduction, in the imaging or genetic domain, is often necessary. Dimensionality-reduction techniques can group or cluster these large sets of variables (dimensions) in either domain; approaches guided by a priori knowledge might prove useful as the field advances [130]. Each multivariate approach has particular advantages and limitations. Data-driven multivariate techniques, such as ICA, in particular, can lead to sample-specific solutions that are difficult to replicate in independent datasets. The large datasets now available through collaborative efforts provide the opportunity to assess and compare the utility of these approaches [37]; on the other hand, larger datasets can also overcome the need for dimensionality-reduction methods if the sample sizes prove sufficient for mass univariate testing.

## Emerging pathways

Understanding the pathways involved in brain development, structure, function, and plasticity will ultimately lead to an improved ability to navigate neuropsychiatric disease pathophysiology. Investigation of the signatures of selection affecting neuropsychiatric, behavioral, and brain phenotypes have indicated both recent and evolutionarily conserved polygenic adaptation, with enrichment in genes affecting neurodevelopment or immune pathways [131] (Table 1). Annotation of the loci associated with subcortical brain volumes has already identified an enrichment of genes related to neurodevelopment, synaptic signaling, ion transport and storage, axonal transport, neuronal apoptosis, and neural growth and differentiation [4, 15, 46] (Table 1). Studies have also implicated pleiotropy (a single locus that affects multiple phenotypes) amongst these loci [46]. Furthermore, many of the associated neurodevelopmental genes are conserved across species, providing a foundation for translational research in imaging genomics [46].

**Table 1:** Emerging pathways in neuroimaging-genomics studies

Neural phenotype	Clinical manifestations	Enriched pathways	Examples of studies that identified these associated pathways in humans
Subcortical brain volumes	On average, hippocampal volumes are smaller in patients with depression [176], bipolar disorder [177], and schizophrenia [178] compared with healthy controls	• Neurodevelopment • Synaptic signaling • Ion transport and storage • Axonal transport • Neuronal apoptosis • Neural growth • Neural differentiation • Immune pathways	Hibar et al. 2015, 2017 [4, 44]
Brain connectivity	Brain white matter microstructure is disrupted globally in schizophrenia [179]	• ATP synthesis and metabolism • Axon guidance • Fasciculation during development	Fornito et al. 2015 [133]
			Vértes et al. 2016 [134]
Transcriptional profiles	Transcription factor EGR1 significantly downregulated in brains of schizophrenic patients compared with controls [180]	• Ion channels • Synaptic activity • ATP metabolism	Wang et al. 2015 [136]
			Richiardi et al. 2015 [137]

Advances in our concepts of brain connectivity can provide a useful framework for further integration of imaging and genomics data. Recent work has emphasized that hubs of neural connectivity are associated with transcriptional differences in genes affecting ATP synthesis and metabolism in mice [132], consistent with their high energy demands [132]. Analogous findings have been found in humans [133, 134]. Studies of the transcriptome and the metabolome, now curated by efforts such as the Allen Brain atlas [135], increasingly allow study of issues such as the relationship between resting-state functional connectivity and gene-expression profiles, with early work indicating enrichment in hubs of genes related to ion channels, synaptic activity, and ATP metabolism [136, 137].

## Key considerations in imaging-genomic analyses

While imaging genomics has great potential, the limitations associated with both genetic [57, 138] and imaging [139] studies, as well as some unique concerns, deserve consideration. Here we discuss three important issues, namely (i) possible confounders of heritability estimates in imaging measures, (ii) the necessity of methodological harmonization for cross-site collaborations, and (iii) accounting for the multiple testing burden.

Environmental, physiological, and demographic influences can affect heritability estimates and measurements of brain-related features [72, 73, 140]. Most psychiatric disorders produce subtle changes in brain phenotypes and multiple potential confounding factors might obscure disease-related effects, limiting their utility as endophenotypes. Examples of such potential factors include motion [141, 142] and dehydration [143, 144], to name a few. Differences in data acquisition and analysis types might also contribute to variation between studies [145], particularly for small structures and grey-matter volumes [146–148]. These potential confounding factors can, however, be included as covariates and adjusted. This approach was used, for example, to control for the effects of height in the largest imaging-genetics meta-analysis of intracranial volume [45]. The distribution of these covariates can also be balanced between cases and controls. Furthermore, potential confounders can be mitigated by investigating healthy individuals only or a single ethnic group, sex, or age group, for example [149]. However, healthy individuals with certain genotypes might be more susceptible to certain confounding factors, such as smoking, which could lead to spurious associations [139].

Furthermore, caution should be taken when interpreting results from fMRI studies, owing to the dependence on quality of both the control and task of interest [150]. These tasks should improve sensitivity and power of genetic effects, adequately stimulate regions of interest, be appropriate for the disorder of interest, reliably evoke reactions amongst individuals, and highlight variability between them [150–152]. Resting-state fMRI studies also require consideration as these might be experienced differently between patients and controls [153]. Studies of unaffected siblings could be beneficial to minimize the potential confounders of disease on brain measures [154]. Meta-analytical approaches need to take the comparability of tasks into account, as apparently slight differences can considerably confound associations [155]. ENIGMA, for example, attempts to reduce these effects through predetermined protocols and criteria for study inclusion [37].

There is often a need to account for multiple testing in imaging genomics beyond that which is done in genetics alone. This is an important issue to emphasize [149, 156]. Studies performing a greater number of tests, especially genome-wide analyses [157] and multimodal and multivariate approaches [130], might require more-stringent corrections. Approaches to reduce the dimensions of these datasets are being developed and include the use of imaging or genetic clusters [66, 158–162] and machine learning methods [163]. However, replication studies and meta-analyses of highly harmonized studies remain the most reliable method for reducing false-positive associations [164].

## Conclusions and future directions

The field of imaging genomics is moving forward in several research directions to overcome the initial lack of reproducible findings and to identify true findings that can be used in clinical practice. First, well-powered hypothesis-free genome-wide approaches remain key. Research groups are now routinely collaborating to ensure adequate power to investigate CNVs and epigenetic, gene–gene, and gene–environment interactions. Second, advances in both imaging and genetic technologies are being used to refine the brain–gene associations; next-generation sequencing (NGS) approaches now allow for more-in-depth investigation of the genome and deeper sequencing (whole-exome and genome); and more-refined brain mapping will ideally allow the field to localize genetic effects to specific tissue layers and subfields as opposed to global structural volumes. Third, replication attempts are crucial, and investigations in various population groups might validate associations and discover new targets that lend further insights into the biological pathways involved in these traits. Finally, specific initiatives to integrate neurogenetics and neuroimaging data for translation into clinical practice are being routinely advocated. These might include efforts in translational neuroscience [165], a systems-biology perspective [16, 166–168], and longitudinal data collection in community and clinical contexts [169].

Current psychiatric treatments have important limitations. First, many patients are refractory to treatment. For example, only approximately 60% of patients with depression achieve remission after either, or a combination of, psychotherapy and pharmacotherapy [170]. Second, clinical guidelines often focus on the “typical” patient, with relatively little ability to tailor individual treatments to the specific individual. Such limitations speak to the complex nature of the brain and of psychiatric disorders, and the multiple mechanisms that underlie the relevant phenotypes and dysfunctions. [20]. In order to progress into an era of personalized medicine, addressing the unique environmental exposures and genetic makeup of individuals [171], further efforts to improve statistical power and analyses are needed.

Ultimately, understanding the mechanisms involved in associated and interconnected pathways could lead to identification of biological markers for more-refined diagnostic assessment and new, more effective, and precise pharmacological targets [20, 171]. These goals can be fostered through continued efforts to strengthen collaboration and data sharing. Indeed, such efforts have led to a growing hope that findings in imaging genomics might well be translated into clinical practice [166–168]. The studies reviewed here provide important initial insights into the complex architecture of brain phenotypes; ongoing efforts in imaging genetics are well positioned to advance our understanding of the brain and of the underlying neurobiology of complex mental disorders, but, at the same time, continued and expanded efforts in neuroimaging genomics are required to ensure that this work has clinical impact.

## Abbreviations

ADNI: Alzheimer's Disease Neuroimaging Initiative
ATP: Adenosine triphosphate
CHARGE: Cohorts for Heart and Aging Research in Genomic Epidemiology
CNV: Copy number variation
DTI: Diffusion-tensor imaging
ENIGMA: Enhancing Neuro Imaging Genetics through Meta-analysis
fMRI: Functional magnetic resonance imaging
GWAS: Genome-wide association study
GxE: Gene–environment interaction
ICA: Independent component analysis
MDD: Major depressive disorder
MRI: Magnetic resonance imaging
PRS: Polygenic risk scoring
RDoC: Research Domain Criteria project
